# Association of diabetes and diabetes treatment with the host response in critically ill sepsis patients

**DOI:** 10.1186/s13054-016-1429-8

**Published:** 2016-08-06

**Authors:** Lonneke A. van Vught, Brendon P. Scicluna, Arie J. Hoogendijk, Maryse A. Wiewel, Peter M. C. Klein Klouwenberg, Olaf L. Cremer, Janneke Horn, Peter Nürnberg, Marc M. J. Bonten, Marcus J. Schultz, Tom van der Poll

**Affiliations:** 1 Center for Experimental and Molecular Medicine, Academic Medical Center, University of Amsterdam, Meibergdreef 9, Room G2-130, 1105 AZ Amsterdam, The Netherlands; 2 the Center for Infection and Immunity, Academic Medical Center, University of Amsterdam, Amsterdam, The Netherlands; 3 Department of Intensive Care Medicine, University Medical Center Utrecht, Utrecht, The Netherlands; 4 Department of Medical Microbiology, University Medical Center Utrecht, Utrecht, The Netherlands; 5 Julius Center for Health Sciences and Primary Care, University Medical Center Utrecht, Utrecht, The Netherlands; 6 Department of Intensive Care, Academic Medical Center, University of Amsterdam, Amsterdam, The Netherlands; 7 Cologne Center for Genomics (CCG), University of Cologne, Cologne, Germany; 8 Cologne Excellence Cluster on Cellular Stress Responses in Aging-Associated Diseases (CECAD), University of Cologne, Cologne, Germany; 9 Center for Molecular Medicine Cologne (CMMC), University of Cologne, Cologne, Germany; 10 Laboratory of Experimental Intensive Care and Anesthesiology, Academic Medical Center, University of Amsterdam, Amsterdam, The Netherlands; 11 Division of Infectious Diseases, Academic Medical Center, University of Amsterdam, Amsterdam, The Netherlands

**Keywords:** Diabetes, Critically ill, Intensive care unit, Sepsis, Host response, Biomarker, MARS study

## Abstract

**Background:**

Diabetes is associated with chronic inflammation and activation of the vascular endothelium and the coagulation system, which in a more acute manner are also observed in sepsis. Insulin and metformin exert immune modulatory effects. In this study, we aimed to determine the association of diabetes and preadmission insulin and metformin use with sepsis outcome and host response.

**Methods:**

We evaluated 1104 patients with sepsis, admitted to the intensive care unit and stratified according to the presence or absence of diabetes mellitus. The host response was examined by a targeted approach (by measuring 15 plasma biomarkers reflective of pathways implicated in sepsis pathogenesis) and an unbiased approach (by analyzing whole genome expression profiles in blood leukocytes).

**Results:**

Diabetes mellitus was not associated with differences in sepsis presentation or mortality up to 90 days after admission. Plasma biomarker measurements revealed signs of systemic inflammation, and strong endothelial and coagulation activation in patients with sepsis, none of which were altered in those with diabetes. Patients with and without diabetes mellitus, who had sepsis demonstrated similar transcriptional alterations, comprising 74 % of the expressed gene content and involving over-expression of genes associated with pro-inflammatory, anti-inflammatory, Toll-like receptor and metabolic signaling pathways and under-expression of genes associated with T cell signaling pathways. Amongst patients with diabetes mellitus and sepsis, preadmission treatment with insulin or metformin was not associated with an altered sepsis outcome or host response.

**Conclusions:**

Neither diabetes mellitus nor preadmission insulin or metformin use are associated with altered disease presentation, outcome or host response in patients with sepsis requiring intensive care.

**Electronic supplementary material:**

The online version of this article (doi:10.1186/s13054-016-1429-8) contains supplementary material, which is available to authorized users.

## Background

Sepsis is characterized by a detrimental unbalanced host response to an infection, resulting in damage to tissues and dysfunction of organs [1]. The outcome of sepsis is influenced by elements associated with the causative pathogen, the primary source of the infection and the host. With respect to the latter, genetic composition, age and comorbidities are considered to contribute [1].

Diabetes mellitus is one of the most common comorbidities in patients with sepsis [2]. Diabetes mellitus is associated with increased susceptibility to infection and sepsis [3], likely due, at least in part, to compromised immune responses, such as adhesion, chemotaxis, phagocytosis and bacterial killing by immune cells [3, 4]. Diabetes mellitus can be accompanied by chronic organ dysfunction together with a state of low-grade chronic inflammation and activation of the vascular endothelium and the coagulation system [4–6], pathological alterations that in a more acute way also feature in patients with sepsis [1]. Nonetheless, several studies have reported that diabetes does not change the mortality of sepsis [7–10]. Knowledge of the possible influence of diabetes mellitus on the host response to severe sepsis is limited. One investigation reported similar cytokine and procoagulant responses in critically ill patients with sepsis with and without pre-existing diabetes mellitus [9], while another study reported elevated levels of endothelial cell activation markers in patients with diabetes mellitus and septic shock, relative to patients without diabetes mellitus [11].

The primary objective of the present study was to provide more insight into the association between pre-existing diabetes mellitus and the host response to sepsis. To this end, we studied the host response in a large prospective cohort of well-documented critically ill patients with sepsis stratified according to the presence or absence of diabetes mellitus, using both a targeted approach (by measuring 15 plasma biomarkers reflective of pathways implicated in sepsis pathogenesis) and an unbiased approach (by analyzing the whole genome expression profiles in blood leukocytes). Additionally, considering that the common anti-diabetic drugs insulin [12–15] and metformin [16–18] can exert immune modulatory effects, the secondary objective of this study was to determine whether patients with diabetes mellitus treated with either of these medications presented with an altered host response to sepsis.

## Methods

### Study design, setting and patient identification

This study was conducted as part of the Molecular Diagnosis and Risk Stratification of Sepsis (MARS) project, a prospective observational study in the mixed intensive care units (ICUs) of two tertiary teaching hospitals (Academic Medical Center in Amsterdam and University Medical Center in Utrecht) (ClinicalTrials.gov identifier NCT01905033) [19–21]. Consecutive patients above 18 years of age admitted between January 2011 and July 2013 with an expected length of stay longer than 24 hours were included via an opt-out method approved by the medical ethical committees of the participating hospitals. All patient data were encrypted for privacy reasons and no separate ethical approval was required for sub-studies such as the one described here. Analyses were limited to the first 2.5 years of the MARS project, because host response measurements were restricted to this period for financial reasons. For every admitted patient the plausibility of an infection was assessed using a 4-point scale (ascending from none to possible, probable or definite) using the Center for Disease Control and Prevention and International Sepsis Forum consensus definitions, making use of all clinical, radiological and microbiological data, as previously described in detail [19]. Sepsis was defined as the presence of infection diagnosed within 24 hours after ICU admission with a probable or definite likelihood, accompanied by at least one additional parameter as described in the 2001 International Sepsis Definitions Conference [22]. Dedicated research physicians prospectively collected demographic data, details of comorbidities (including the Charlson comorbidity index [23]), and daily clinical data and severity scores, including Acute Physiology and Chronic Health Evaluation (APACHE) IV [24] and Sequential Organ Failure Assessment (SOFA) scores (the central nervous system was excluded) [25].

Diabetes mellitus was defined as known history of type I or type II diabetes mellitus prior to ICU admission or by the use of oral anti-diabetic medication or insulin as chronic medication. The timing of the last dose of insulin or metformin prior to ICU admission was not registered; metformin was stopped after ICU admission. Cardiovascular insufficiency was defined as a medical history of congestive heart failure, chronic cardiovascular disease, peripheral vascular disease or cerebrovascular disease. Malignancy was defined by medical history of either non-metastatic solid tumor, metastatic malignancy or hematologic malignancy. Patients with a history of chronic renal insufficiency or with chronic intermittent hemodialysis or continuous ambulatory peritoneal dialysis were marked as renal insufficient [21].

Specific organ failure was defined by a SOFA score ≥3, except for cardiovascular failure for which a score ≥1 was used (the central nervous system was excluded) [26]. Shock was defined by the use of vasopressors (noradrenaline) for hypotension in a dose >0.1 μg/kg/min during at least 50 % of the ICU day. Acute kidney injury and acute lung injury were defined using strict pre-set criteria [27, 28]. ICU-acquired complications were defined as events that started 2 days or more after ICU admission. The Municipal Personal Records Database was consulted to calculate survival after ICU admission. Patients readmitted to the same ICU or transferred from other ICUs were excluded, except when patients were referred to one of the study centers on the day of admission.

### Plasma protein assays

Daily (on admission and at 6 a.m. thereafter) EDTA anti-coagulated leftover plasma harvested from blood obtained for regular patient care was stored within 4 hours after blood draw at -80 °C. Measurements were done in EDTA anti-coagulated plasma. Tumor necrosis factor (TNF)-α, interleukin (IL)-6, IL-8, IL-1β, IL-10, IL-13, interferon-γ, soluble E-selectin, soluble intercellular adhesion molecule (ICAM)-1 and fractalkine were measured by FlexSet cytometric bead array (BD Biosciences, San Jose, CA, USA) using FACS Calibur (Becton Dickenson, Franklin Lakes, NJ, USA). Matrix metalloproteinase (MMP)-8, angiopoietin-1, angiopoietin-2, protein C, antithrombin (all R&D systems, Abingdon, UK) and D-dimer (Procartaplex, eBioscience, San Diego, CA, USA) were measured by Luminex multiplex assay using BioPlex 200 (BioRad, Hercules, CA, USA). C-reactive protein (CRP) was determined by immunoturbidimetric assay (Roche diagnostics), prothrombin time (PT) and activated partial thromboplastin time (aPTT) by using a photometric method with Dade Innovin Reagent or by Dade Actin FS Activated PTT Reagent, respectively (both Siemens Healthcare Diagnostics). Normal values were obtained in plasma from 27 age-matched and gender-matched healthy volunteers, included after providing written informed consent, with the exception of CRP, PT and aPTT (routine laboratory reference values).

### Blood gene expression microarrays

Whole blood was collected in PAXgene™ tubes (Becton-Dickinson, Breda, the Netherlands) within 24 hours after ICU admission. Total RNA was isolated using the PAXgene blood mRNA kit (Qiagen, Venlo, the Netherlands) in combination with QIAcube automated system (Qiagen, Venlo, the Netherlands), according to the manufacturer’s instructions. RNA (RNA integrity number >6.0) was processed and hybridized to the Affymetrix Human Genome U219 96 array and scanned using the GeneTitan instrument at the Cologne Center for Genomics (CCG), Cologne, Germany, as described by the manufacturer (Affymetrix). Raw data scans (.CEL files) were read into the R language and environment for statistical computing (version 2.15.1; R Foundation for Statistical Computing, Vienna, Austria; http://www.R-project.org/).

Pre-processing and quality control was performed using the Affy package version 1.36.1. Array data were background-corrected by Robust Multi-array Average, quantile-normalized and summarized by medianpolish using the expresso function (Affy package). The resultant 49,386 log-transformed probe intensities were filtered by means of a 0.5 variance cutoff using the genefilter method [29] to recover 24,646 expressed probes in at least one sample. The occurrence of non-experimental chip effects was evaluated by means of the Surrogate Variable Analysis (R package version 3.4.0) and corrected by the empirical Bayes method ComBat [30]. The non-normalized and normalized MARS gene expression data sets are available at the Gene Expression Omnibus public repository of the NCBI [GEO:GSE65682].

The 24,646 probes were assessed for differential abundance across healthy subject and patient samples by means of the limma method (version 3.14.4) [31]. Supervised analysis (comparison between predefined groups) was performed by moderated *t* statistics. Throughout, significance was defined using the Benjamini-Hochberg (BH) multiple comparison adjusted probabilities, correcting for the 24,646 probes (false discovery rate <5 %). Ingenuity Pathway Analysis (Ingenuity Systems IPA, www.ingenuity.com) was used to identify the associated canonical signaling pathways stratifying genes by over-expressed and under-expressed patterns. The Ingenuity gene knowledgebase was selected as reference and human species specified. All other parameters were left at default. The significance of association was assessed using Fisher’s exact test.

### Statistical analysis

All data distributions were tested for normality using the Shapiro-Wilk test and histogram plots. The Mann-Whitney *U* test or Kruskal-Wallis test was used to analyze continuous nonparametric data, presented as median and interquartile range (IQR, 25^th^ and 75^th^ percentiles). Continuous parametric data, presented as numbers (percentages) or as means ± standard deviation (SD), were analyzed using Student’s *t* test or analysis of variance when appropriate. All categorical data were analyzed using the chi square test. As the biomarker data were not normally distributed, the Kruskal-Wallis test was used to analyze non-parametric data. A multivariable cox proportional hazard model was used to determine the association between diabetes mellitus and mortality. The covariables included in the model were BMI, patient age, gender, cardiovascular insufficiency, renal insufficiency and hypertension. A sensitivity analysis was conducted, correcting for the APACHE IV score.

All data were analyzed using R studio built under R version 3.0.2 (R Core Team 2013, Vienna, Austria) [32]. The R package *survival* was used for the survival analysis. Multiple-comparison-adjusted (BH) *p* values <0.05 were used to define the significance of plasma biomarkers.

## Results

### Patients, sepsis presentation and outcome

During the 2.5-year study period 1483 ICU admissions with sepsis were screened for eligibility; after exclusion of 250 patients (16.9 %) who were readmitted and 129 patients (8.7 %) who were transferred from other ICUs, 1104 patients remained for study inclusion, of whom 241 (21.8 %) had a medical history of diabetes mellitus. Patients with diabetes mellitus were older, had a higher BMI, a higher modified Charlson Comorbidity Index (calculated without the contribution of diabetes mellitus) and were admitted with more chronic comorbidities such as cardiovascular compromise, hypertension and renal insufficiency (Table 1). Gender and race did not differ between groups. Insulin was noted as the medication for chronic disease in 54.8 % of patients with diabetes mellitus, and metformin was used in 47.3 % of the patients with diabetes mellitus.

**Table 1:** Baseline characteristics, clinical course and outcome of critically ill patients with sepsis with or without diabetes mellitus

	Diabetes mellitus	No diabetes mellitus	*P* value
Patients, *n* (%)	241 (21.8 %)	863 (78.2 %)	
Demographic data			
Age, mean (SD), years	66.0 (11.6)	59.8 (15.2)	<0.0001
Gender male, *n* (%)	144 (59.8 %)	527 (61.1 %)	0.77
Body mass index mean (SD)	28.9 (7.7)	25.2 (5.4)	<0.0001
Race, white, *n* (%)	202 (83.8 %)	771 (89.3 %)	0.10
Medical admission, *n* (%)	188 (78.0 %)	630 (73.0 %)	0.13
Chronic comorbidity, *n* (%)			
Cardiovascular compromise	103 (42.7 %)	160 (18.5 %)	<0.0001
Chronic obstructive pulmonary disease	37 (15.4 %)	124 (14.4 %)	0.74
Hypertension	129 (53.5 %)	211 (24.4 %)	<0.001
Malignancy	61 (25.3 %)	215 (24.9 %)	0.94
Renal insufficiency	57 (23.7 %)	104 (12.1 %)	<0.001
Modified Charlson Comorbidity Index^a^, median (IQR)	5 (3–6)	4 (2­–5)	<0.0001
Diabetic medication, *n* (%)			
Insulin	133 (55.2 %)		
Metformin	114 (47.3 %)	-	
Glimepiride	22 (9.1 %)	-	
Glibenclamide	1 (0.4 %)	-	
Gliclazide	5 (2.1 %)	-	
Tolbutamide	24 (10.0 %)	-	
Severity of disease on ICU admission			
APACHE IV Score, median (IQR)	82 (66–104)	79 (52–101)	0.03
APACHE APS, median (IQR)	67 (52–87)	67 (50–85)	0.44
SOFA score, median (IQR)	7 (5–9)	7 (5–9)	0.55
Mechanical ventilation, *n* (%)	155 (64.3 %)	601 (69.6 %)	0.12
Organ failure, *n* (%)	202 (83.8 %)	721 (83.5 %)	0.48
Shock, *n* (%)	78 (32.4 %)	295 (34.2 %)	0.65
Acute kidney injury, *n* (%)	96 (39.8 %)	308 (35.7 %)	0.26
Acute lung injury, *n* (%)	61 (25.3 %)	219 (25.4 %)	>0.99
Acute myocardial infarction, *n* (%)	7 (2.9 %)	17 (2.0 %)	0.45
Laboratory measurements first 24 hours after ICU admission (peak values), median (IQR)			
Glucose (mmol/L)	12.9 (10.5–16.2)	9.5 (7.9–11.5)	<0.0001
Lactate (mmol/mL)	3.1 (1.8–4.8)	2.5 (1.6–4.9)	0.14
Creatinin (μmol/L	135 (89–197)	109 (71–181)	<0.0001
Source of infection, *n* (%)			
Pulmonary	103 (42.7 %)	430 (49.8 %)	0.07
Abdominal	53 (22.0 %)	188 (21.8 %)	>0.99
Urosepsis	42 (17.4 %)	85 (9.8 %)	0.002
Cardiovascular infection	29 (12.0 %)	124 (14.4 %)	0.41
Skin	14 (5.8 %)	36 (4.2 %)	0.32
Causative pathogens^b^, *n* (%)			
Gram-negative bacteria	141 (58.5 %)	444 (51.4 %)	0.06
Gram-positive bacteria	102 (42.3 %)	386 (44.7 %)	0.55
Fungi/yeasts	26 (10.8 %)	80 (9.3 %)	0.56
Virus	11 (4.6 %)	46 (5.3 %)	0.76
Other	17 (7.1 %)	54 (6.3 %)	0.78
Unknown	31 (12.9 %)	134 (15.6 %)	0.36
Outcome			
ICU length of stay (days), median (IQR)	4 (2–9)	4 (2–10)	0.56
Hospital length of stay (days), median (IQR)	21 (10–40)	23 (11–47)	0.08
Complications, *n* (%)			
None	199 (82.6 %)	702 (81.3 %)	0.70
Acute kidney injury	25 (10.4 %)	69 (8.0 %)	0.30
Acute lung injury	10 (4.1 %)	41 (4.8 %)	0.74
ICU-acquired weakness	13 (5.4 %)	60 (7.0 %)	0.46
Acute myocardial infarction	2 (0.8 %)	8 (0.9 %)	>0.99
ICU-acquired infection	22 (9.1 %)	88 (10.2 %)	0.63
Mortality, *n* (%)			
ICU	47 (19.5 %)	180 (20.9 %)	0.65
Hospital	78 (32.4 %)	270 (31.3 %)	0.83
Day 30	72 (29.9 %)	233 (27.0 %)	0.44
Day 60	90 (37.3 %)	275 (31.9 %)	0.15
Day 90	95 (39.4 %)	308 (35.7 %)	0.32

^a^Modified Charlson Index was calculated without the contribution of diabetes mellitus. ^b^Percentages of causative pathogens were calculated using the total number of infections as denominator. As multiple pathogens could be assigned as causative, percentages >100 % may occur. *APACHE* Acute Physiology and Chronic Health Evaluation, *APS* Acute Physiology Score, *ICU* intensive care unit, *IQR* interquartile range

Disease severity on ICU admission was comparable in patients with and without known diabetes mellitus. Although the APACHE IV score was higher in diabetes mellitus patients, the difference compared to patients without diabetes mellitus was driven by differences in age and comorbidities, as the Acute Physiology Score (APS) was comparable between groups. Patients with known diabetes mellitus were more often admitted with urosepsis (17.4 % versus 9.8 % in patients without diabetes mellitus, *p* = 0.002); other sources of infection were not different between groups. Causative pathogens were comparable in patients with and without diabetes mellitus, although there was a trend towards more gram-negative infections in patients with history of diabetes mellitus (Table 1).

Patients with and without diabetes mellitus did not differ in ICU or hospital length of stay, or development of ICU-acquired complications (Table 1). In addition, mortality did not differ between groups during the period up to 90 days after ICU admission (Table 1). Diabetes mellitus was also not associated with increased risk of mortality at 90 days after correction for baseline differences in BMI, age, gender, cardiovascular insufficiency, renal insufficiency and hypertension (hazard ratio (HR) 0.90, 95 % CI 0.69, 1.15), nor did they differ when corrected for APACHE IV score (HR 1.02, 95 % CI 0.81, 1.29).

### Plasma biomarkers reflective of host response pathways implicated in sepsis pathogenesis

**Fig. 1:**
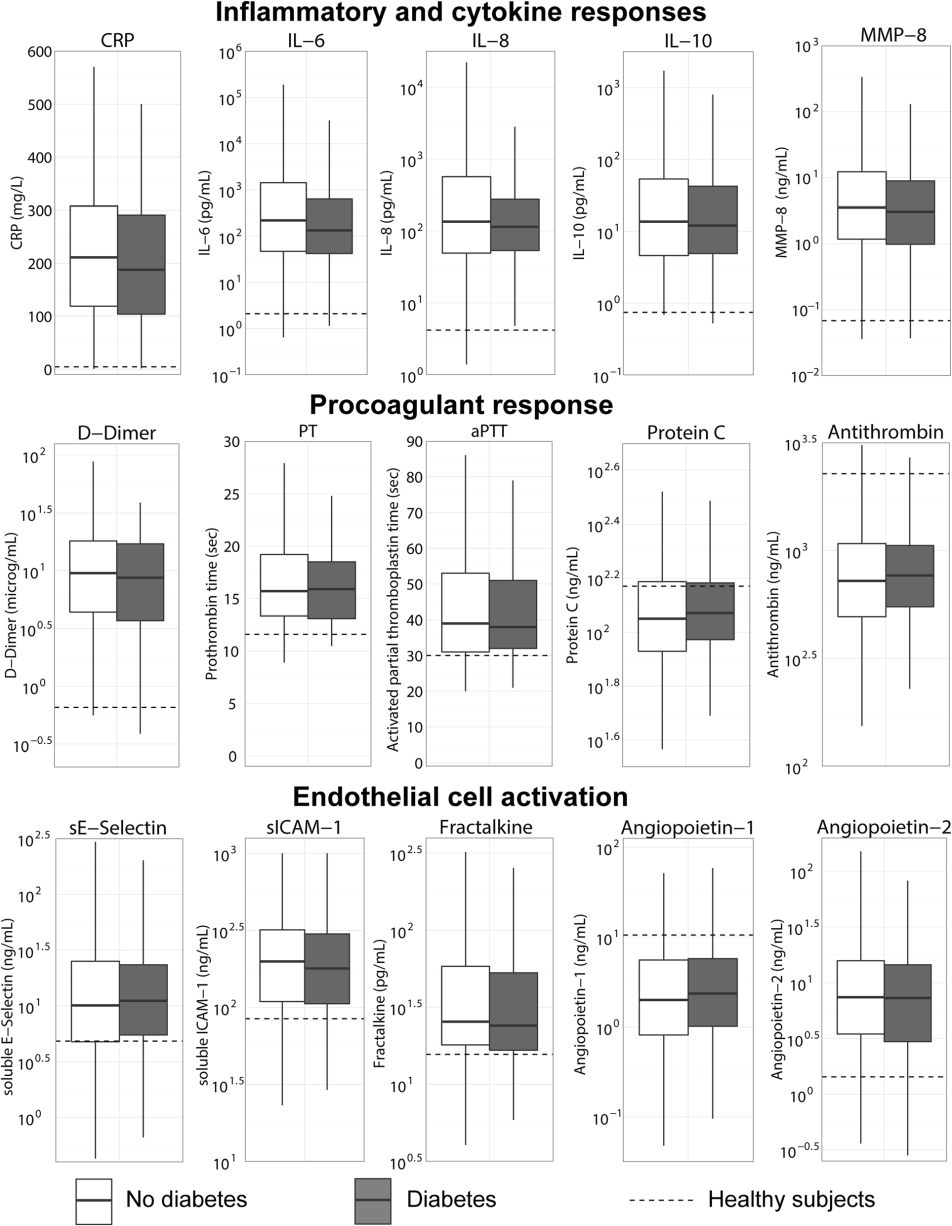
Plasma biomarkers reflective of host response pathways implicated in sepsis pathogenesis on ICU admission in sepsis patients with and without diabetes mellitus. *Boxes* show median and lower and upper quartiles (*solid horizontal lines*) with their respective 1.5 IQR (*whiskers*) (as specified by Tukey). *Dotted lines* indicate median values obtained in 27 healthy age-matched subjects. *CRP* C-reactive protein, *IL* interleukin, *ICAM* intercellular adhesion molecule, *MMP* matrix metalloproteinase, *PT* prothrombin time, *APT* activated partial thromboplastin time

Relative to healthy controls, patients with sepsis had increased acute phase protein response (elevated plasma CRP concentrations), profound activation of the cytokine network (elevated plasma levels of IL-6, IL-8 and IL-10), the vascular endothelium (elevated plasma concentrations of soluble E-selectin, soluble ICAM-1, fractalkine and angiopoietin-2, and reduced levels of angiopoietin-1) and the coagulation system (elevated D-dimer levels, prolonged PT and aPTT, and reduced levels of the anticoagulant proteins protein C and antithrombin), both on ICU admission (Fig. 1) and on days 2 and 4 (Additional file 1: Table S1). Systemic inflammation was further demonstrated by elevated levels of MMP-8. None of these responses differed between patients with and without diabetes mellitus. The plasma concentrations of TNF-α, interferon-γ, IL-1β and IL-13 were undetectable or very low in the vast majority of patients and were not different between groups (data not shown).

### Blood leukocyte transcriptome analysis

**Fig. 2:**
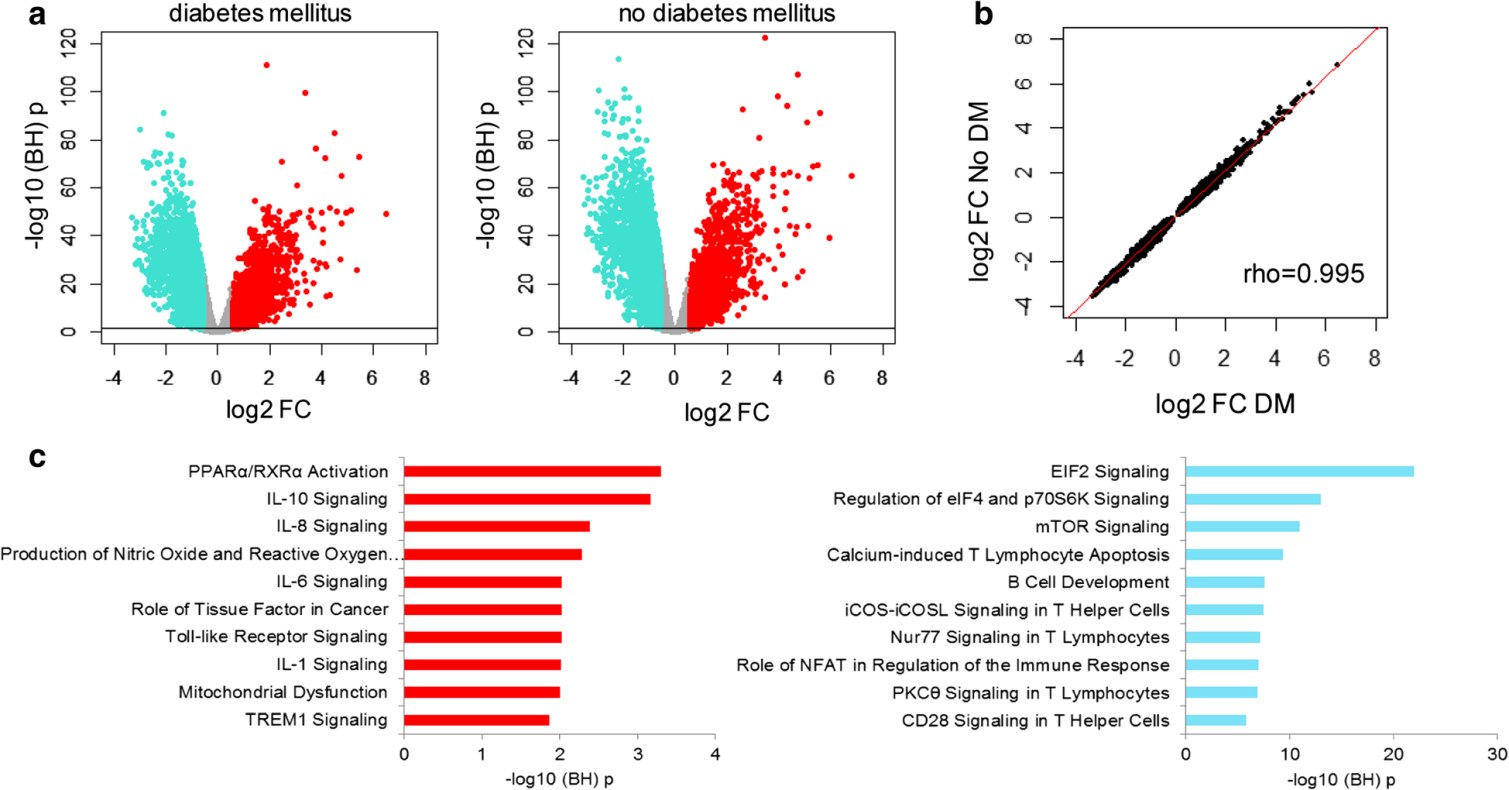
Blood transcriptional profiling of patients with sepsis with and without diabetes mellitus (*DM*). **a** Volcano plot representation of differentially expressed genes of whole-blood leukocytes in sepsis patients discordant for diabetes mellitus, each compared to healthy controls. *Red dots* denote over-expressed genes and *blue dots* denote under-expressed genes. **b** Spearman correlation analysis of the transcriptional alterations in patients with sepsis with or without diabetes mellitus revealed almost perfect correlation. *rho* Spearman’s correlation coefficient, fold change (*FC*). **c** Ingenuity pathway analysis of commonly over-expressed (*red bars*) and under-expressed (*turquoise bars*) genes. –log10 (Benjamini-Hochberg (*BH*)) *p* value negative log_10_-transformed corrected for multiple comparisons

Using an unbiased approach, we compared the blood leukocyte transcriptome of patients with diabetes mellitus (*n* = 108) and those without diabetes mellitus (*n* = 382). This analysis comprised the subgroup of patients enrolled during the first 1.5 years of this study (Additional file 2: Table S2). Demographic differences and differences in comorbidities between patients with and without diabetes mellitus were similar to those found in the complete cohort. Likewise, disease severity at presentation and outcomes did not differ between patients with and without diabetes mellitus. At first, the genome-wide blood gene expression profiles of these sepsis patients were compared to 42 healthy controls. Patients both with and without diabetes mellitus had strong transcriptional alterations (Fig. 2a), and 74 % of the expressed gene content was significantly altered in both sepsis groups, with no differences between patients with sepsis with and without diabetes mellitus (Fig. 2b). Biological pathway analysis revealed commonly over-expressed genes associated with typical pro-inflammatory, anti-inflammatory, Toll-like receptor and metabolic signaling pathways (Fig. 2c). Common under-expressed genes were associated with a variety of T cell signaling pathways and translation (EIF2) and metabolic (mTOR) signaling pathways (Fig. 2c).

### Association with insulin and metformin treatment

To obtain insight into a possible effect of chronic insulin treatment on the host response to sepsis in patients with diabetes mellitus, we first determined differences in the presentation and outcome of patients with diabetes mellitus who were on insulin therapy prior to ICU admission and those who were not (Table 2). Relative to patients with diabetes mellitus who were not on chronic insulin therapy, patients treated with insulin were younger, less frequently male and had less cardiovascular insufficiency, less malignancy, and more renal insufficiency. Among the patients treated with insulin, 36.1 % also received oral anti-diabetic medication.

**Table 2:** Baseline characteristics, clinical course and outcome of patients with diabetes mellitus and sepsis, who were stratified according insulin or metformin use prior to ICU admission

	Diabetes mellitus treated with insulin	Diabetes mellitus not treated with insulin	*P* value	Diabetes mellitus treated with metformin	Diabetes mellitus not treated with metformin	*P* value
Patients, *n* (%)	133 (55.2 %)	108 (44.8 %)		114 (47.3 %)	127 (52.7 %)	
Demographic data						
Age mean (SD), years	64.3 (12.5)	68.1 (10.0)	<0.01	67.0 (10.4)	65.1 (12.6)	0.19
Gender male, *n* (%)	71 (53.4 %)	73 (67.6 %)	0.04	78 (68.4 %)	66 (52.0 %)	0.01
Body mass index mean (SD)	28.9 (8.5)	28.9 (6.7)	0.98	29.0 (7.7)	28.8 (7.9)	0.86
Race, white, *n* (%)	115 (86.5 %)	87 (80.6 %)	0.45	97 (85.1 %)	105 (82.7 %)	0.66
Medical admission, *n* (%)	104 (78.2 %)	84 (77.8 %)	>0.99	92 (80.7 %)	96 (75.6 %)	0.37
Chronic comorbidity, *n* (%)						
Cardiovascular compromise	49 (36.8 %)	54 (50.0 %)	0.05	48 (42.1 %)	55 (43.3 %)	0.89
COPD	22 (16.5 %)	15 (13.9 %)	0.59	16 (14.0 %)	21 (16.5 %)	0.59
Hypertension	66 (49.6 %)	63 (58.3 %)	0.20	65 (57.0 %)	64 (50.4 %)	0.34
Malignancy	25 (18.8 %)	36 (33.3 %)	0.02	32 (28.1 %)	29 (22.8 %)	0.38
Renal insufficiency	41 (30.8 %)	16 (14.8 %)	<0.01	10 (8.8 %)	47 (37.0 %)	<0.001
Modified Charlson Comorbidity Index^a^	4 (3–6)	5 (4–6)	0.01	5 (3–6)	5 (3–6)	0.85
Diabetic medication, *n* (%)						
Insulin	132 (100.0 %)	-		44 (38.6 %)	88 (69.3 %)	<0.001
Metformin	44 (33.1 %)	70 (64.8 %)	<0.001	114 (100.0 %)	-	
Glimepiride	5 (3.8 %)	17 (15.7 %)	<0.01	18 (15.8 %)	4 (3.1 %)	<0.001
Glibenclamide	-	1 (0.9 %)	0.47	1 (0.9 %)	-	
Gliclazide	2 (1.5 %)	3 (2.8 %)	0.66	2 (1.8 %)	3 (2.4 %)	>0.99
Tolbutamide	3 (2.3 %)	21 (19.4 %)	<0.001	8 (7.0 %)	16 (12.6 %)	0,18
Any oral anti-diabetic medication	48 (36.1 %)	89 (82.4 %)	<0.001	114 (100.0 %)	23 (18.1 %)	<0.0001
Severity of disease on ICU admission						
APACHE IV score, median (IQR)	82 (67–106)	83 (65–103)	0.97	83 (64–106)	82 (68–104)	0.62
APACHE APS, median (IQR)	68 (52–87)	65 (51–85)	0.54	66 (50–90)	68 (54–86)	0.56
SOFA score, median (IQR)	7 (6–9)	7 (5–9)	0.58	7 (5–8)	8 (6–10)	0.05
Mechanical ventilation, *n* (%)	79 (59.4 %)	76 (70.4 %)	0.07	70 (61.4 %)	85 (66.9 %)	0.41
Organ failure, *n* (%)	115 (86.5 %)	87 (80.6 %)	0.56	94 (82.5 %)	108 (85.0 %)	0.77
Shock, *n* (%)	46 (34.6 %)	32 (29.6 %)	0.50	28 (24.6 %)	50 (39.4 %)	0.02
Acute kidney injury, *n* (%)	56 (42.1 %)	40 (37.0 %)	0.43	50 (43.9 %)	46 (36.2 %)	0.22
Acute lung injury, *n* (%)	31 (23.3 %)	30 (27.8 %)	0.46	30 (26.3 %)	31 (24.4 %)	0.79
Acute myocardial infarction, *n* (%)	4 (3.0 %)	3 (2.8 %)	>0.99	5 (4.4 %)	2 (1.6 %)	0.25
Laboratory measurements first 24 hours after ICU admission (peak values), median (IQR)						
Glucose (mmol/L)	12.8 (10.8–16.6)	12.9 (10.0–15.5)	0.17	12.5 (10.3–15.5)	13.3 (10.6–16.7)	0.43
Lactate (mmol/mL)	3.2 (2-4.9)	2.9 (1.6–4.5)	0.35	2.7 (1.6–4.5)	3.3 (2–5.1)	0.22
Creatinin (μmol/L)	146 (89–228)	123 (92–173)	0.08	124 (87–170)	151 (95–255)	0.01
Outcome						
Length of ICU stay (days), median (IQR)	4 (2-9)	4 (2-9)	>.99	5 (2-10)	4 (2-9)	0.65
Hospital length of stay (days), median (IQR)	21 (11–44)	21 (9–38)	0.55	22 (11–44)	20 (10–40)	0.44
Complications, *n* (%)						
None	108 (81.2 %)	91 (84.3 %)	0.60	96 (84.2 %)	103 (81.1 %)	0.62
Acute kidney injury	13 (9.8 %)	12 (11.1 %)	0.82	11 (9.6 %)	14 (11.0 %)	0.84
Acute lung injury	7 (5.3 %)	3 (2.8 %)	0.35	4 (3.5 %)	6 (4.7 %)	0.76
ICU-acquired weakness	6 (4.5 %)	7 (6.5 %)	0.56	6 (5.3 %)	7 (5.5 %)	>0.99
Acute myocardial infarction	1 (0.8 %)	1 (0.9 %)	>0.99	2 (1.8 %)	-	0.22
ICU-acquired infection	16 (12.0 %)	6 (5.6 %)	0.13	8 (7.0 %)	14 (11.0 %)	0.39
Mortality, *n* (%)						
ICU	23 (17.3 %)	24 (22.2 %)	0.42	18 (15.8 %)	29 (22.8 %)	0.20
Hospital	43 (32.3 %)	35 (32.4 %)	>0.99	32 (28.1 %)	46 (36.2 %)	0.22
Day 30	41 (30.8 %)	31 (28.7 %)	0.77	31 (27.2 %)	41 (32.3 %)	0.47
Day 60	50 (37.6 %)	40 (37.0 %)	>0.99	38 (33.3 %)	52 (40.9 %)	0.25
Day 90	51 (38.3 %)	44 (40.7 %)	0.79	41 (36.0 %)	54 (42.5 %)	0.30

^a^Modified Charlson Index was calculated without the contribution of diabetes mellitus. *APACHE* Acute Physiology and Chronic Health Evaluation, *APS* Acute Physiology Score, *COPD* chronic obstructive pulmonary disease, *ICU* intensive care unit

**Fig. 3:**
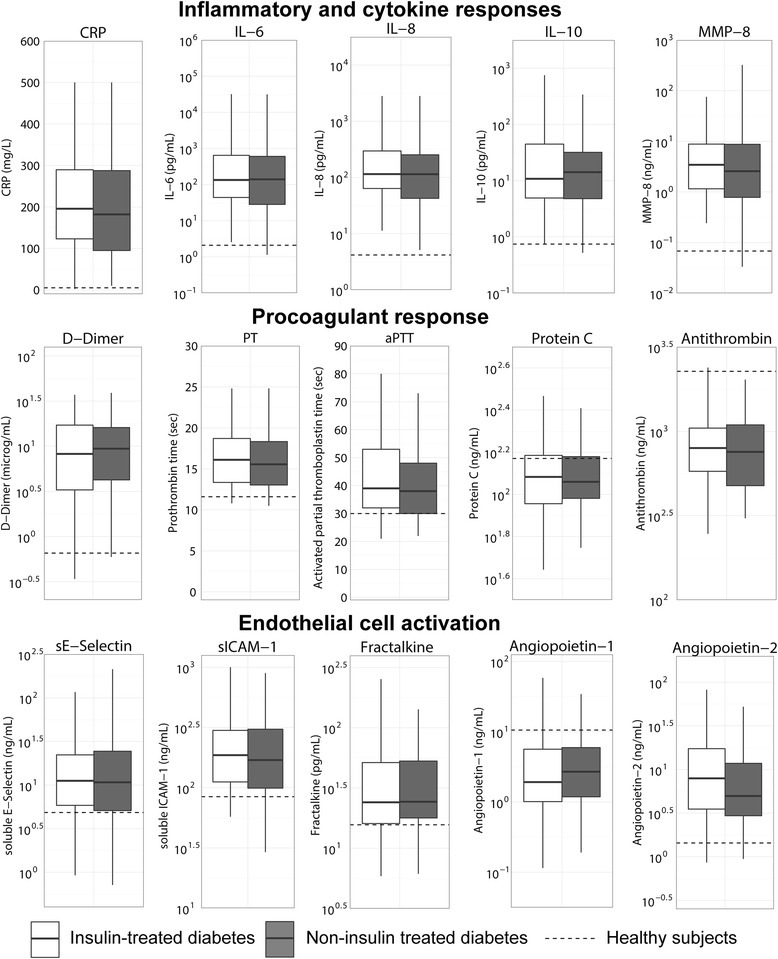
Host response on ICU admission in patients admitted with insulin-treated versus non-insulin-treated diabetes mellitus. *Boxes* show the median, lower quartile and upper quartiles (*solid horizontal lines*) and their respective 1.5 IQR (*whiskers*) (as specified by Tukey). *Dotted lines* indicate median values obtained in 27 healthy age matched subjects. *CRP* C-reactive protein, *IL* interleukin, *ICAM* intercellular adhesion molecule, *MMP* matrix metalloproteinase, *PT* prothrombin time, *APT* activated partial thromboplastin time

The course and mortality of sepsis was similar in patients with diabetes mellitus who were and were not on chronic insulin therapy (Table 2). Chronic insulin treatment did not influence the mortality risk at 90 days when corrected for baseline differences in age, gender, malignancy and renal insufficiency (HR 1.10, 95 % CI 0.72, 1.69) nor when corrected for the APACHE IV score (HR 0.87, 95 % CI 0.58, 1.30). Plasma biomarker levels suggestive of systemic inflammation, activation of the coagulation system and the vascular endothelium did not differ between patients on chronic insulin therapy and those who were not (Fig. 3). Similarly, the blood leukocyte genomic response did not differ between these two groups (Additional file 3: Figure S1A).

**Fig. 4:**
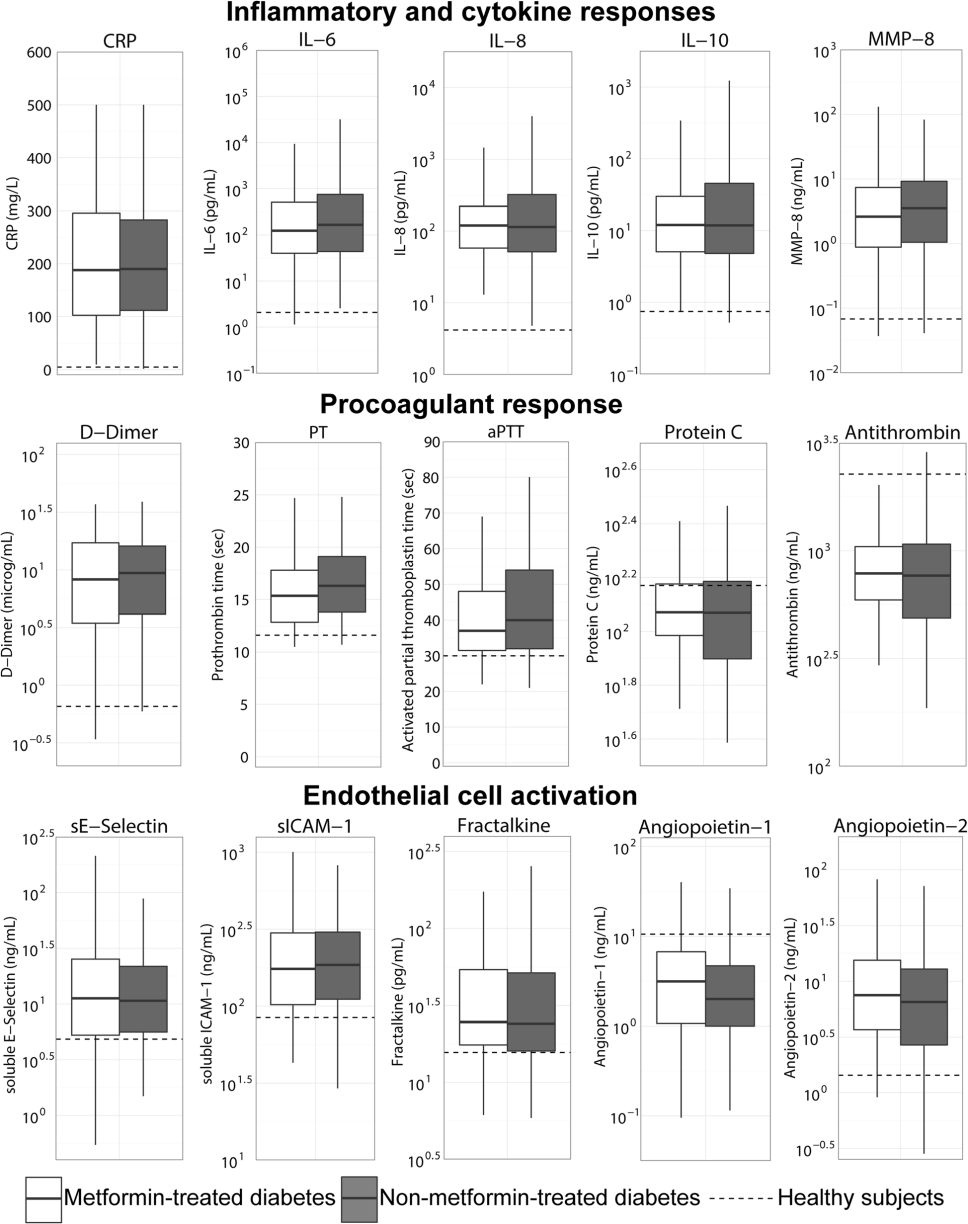
Host response on ICU admission in patients admitted with metformin-treated versus non-metformin-treated diabetes mellitus. Shown are the median, lower and upper quartile (*solid horizonal lines*) and their respective 1.5 IQR (*whiskers*) (as specified by Tukey). *Dotted lines* indicate median values obtained in 27 healthy age matched subjects. *CRP* C-reactive protein, *IL* interleukin, *ICAM* intercellular adhesion molecule, *MMP* matrix metalloproteinase, *PT* prothrombin time, *APT* activated partial thromboplastin time

To obtain insight into a possible influence of prior metformin treatment on the host response to sepsis in patients with diabetes mellitus, we first assessed variance in the presentation and outcome of patients with diabetes mellitus who had received metformin prior to ICU admission and those who had not (Table 2). Relative to diabetes mellitus patients who were not on chronic metformin treatment, patients treated with metformin were more frequently male and had less renal insufficiency. Metformin treatment was not associated with an altered course of disease or the case fatality rate of sepsis (Table 2). Chronic metformin treatment did not influence the mortality risk at 90 days when corrected for baseline differences in age, gender and renal insufficiency (HR 0.75, 95 % CI 0.48, 1.15) nor when corrected for the APACHE IV score (HR 0.81, 95 % CI 0.54, 1.21). The host response to sepsis, as measured by plasma protein markers (Fig. 4) and the whole genome transcriptome in blood leukocytes (Additional file 3: Figure S1B) did not differ between patients with diabetes mellitus who had and had not received prior metformin therapy.

## Discussion

The main finding of this investigation is that sepsis patients with a medical history of diabetes mellitus have a similar host response upon ICU admission when compared with sepsis patients without known diabetes mellitus. In addition, pre-existing diabetes mellitus did not alter sepsis-associated mortality up to 90 days after ICU admission. We also demonstrated that amongst patients with diabetes mellitus two common diabetes mellitus therapies, insulin and metformin, are not associated with a modified host response or sepsis outcome.

Our data on similar case fatality rates of sepsis in critically ill patients with or without a known history of diabetes mellitus are in accordance with previous studies [7, 9, 10]. Notably, however, other investigations have reported increased [33, 34] or decreased [35] sepsis mortality in patients with diabetes mellitus. These latter studies differ from ours in that they did not merely include patients with sepsis in need of intensive care, and as a consequence thereof reported much lower mortality rates than observed in our sepsis ICU population. Together these data suggest that the association between diabetes mellitus and sepsis outcome depends, at least in part, on the (sub)acute severity of disease.

Diabetes mellitus was not associated with altered host responses during sepsis, suggesting that diabetes-mellitus-related low-grade chronic inflammation, activation of the vascular endothelium and the coagulation system [4–6], does not influence acute infection-induced alterations in these pathways. In accordance, a previous study likewise reported comparable markers of coagulation and inflammation in patients with diabetes mellitus and sepsis [9]. Similarly, diabetes mellitus was not associated with altered cytokine or procoagulant responses in patients presenting at the emergency room with community-acquired pneumonia [36].

We measured several plasma biomarkers of endothelial cell activation (soluble E-selectin, soluble ICAM-1 and fractalkine) [37] and barrier function (angiopoietin-1 and angiopoietin-2) [37], neither of which was modified in patients with diabetes mellitus. The vascular endothelium is of great interest in the context of diabetes mellitus and sepsis, because chronic diabetes mellitus can cause a dysfunctional endothelium leading to diabetes-mellitus-related atherosclerosis [38], and upregulation of ICAM-1, vascular cell adhesion molecule-1 (VCAM-1) and E-selectin, resulting in increased adherence of neutrophils [39]. An earlier investigation reported endothelial cell activation plasma markers in patients presented to the emergency department with clinically suspected infection [11]. In this cohort soluble ICAM-1 and VCAM-1 did not differ between patients with and without diabetes mellitus; however, plasma-soluble E-selectin and soluble fms-like tyrosine kinase-1 were higher in patients with diabetes mellitus; notably, unlike our investigation, diabetes mellitus patients presented with more severe disease when compared with patients who did not have diabetes mellitus, possibly confounding the interpretation of plasma biomarker measurements [11].

Similarities in patients with sepsis who did and did not have diabetes mellitus were not exclusive to plasma biomarkers, as we also observed no differences in their blood leukocyte transcriptomes. Almost identical alterations were evident in both groups of patients as compared to healthy controls; alterations that were strongly attuned to a “common host/sickness response” [20]. Over-expression of typical pro-inflammatory, anti-inflammatory and metabolic pathways in parallel with under-expression of a plethora of T cell related pathways were overarching features of the common response. Notably, key cellular metabolic pathways (mitochondrial dysfunction, EIF2 and MTOR signaling) were also similarly altered in patients with and without diabetes mellitus.

Our study is the first to analyze sepsis patients with insulin-treated compared to patients with non-insulin-treated diabetes mellitus, revealing no differences between groups in the presentation or outcome of sepsis, or the host responses. An earlier investigation reported increased disease severity in critically ill patients with insulin-treated diabetes mellitus relative to all other patients on ICU admission [10]. However, this study is difficult to compare with ours, because it was not restricted to patients with sepsis and the comparator group consisted of both patients with diabetes mellitus not treated with insulin and patients without diabetes mellitus [10]. As insulin can modify inflammatory and procoagulant responses independently of glucose levels [12–15] we sought to compare the host response in insulin-treated versus non-insulin-treated patients with diabetes mellitus. Pre-existing insulin treatment did not affect any of the plasma host response biomarkers or the blood genomic response in our cohort, possibly due to the more profound alterations caused by the sepsis response in both groups.

Considering the established anti-inflammatory effects of metformin [16, 17], we compared the presentation and outcome of sepsis and the host responses in metformin-treated and non-metformin-treated patients with diabetes mellitus. Metformin had no effect on the outcome or host response, despite the fact that patients with non-metformin-treated diabetes mellitus were admitted with significantly higher rates of shock. Metformin may decrease mortality in other patient populations. In patients with diabetes mellitus who underwent cardiac surgery, preadmission metformin use was associated with a decreased postoperative morbidity rate, including from infections, and with a substantial decrease in inpatient mortality [40]. In a general population of medical and surgical ICU patients with type II diabetes mellitus, preadmission metformin use was associated with reduced 30-day mortality; however, this was not statistically significant in the subgroup of patients with sepsis [41]. Blood transcriptome data obtained from patients with diabetes mellitus and sepsis, who were or were not treated with metformin suggests no influence of gluconeogenesis antagonism on systemic genomic responses.

Our study has strengths and limitations. It is the first to report on the potential influence of diabetes mellitus on the blood genomic response to sepsis and the first of its size to extensively study the association between treatments for diabetes mellitus and the host response to sepsis. Due to our prospective data collection we present a well-defined sepsis population with detailed clinical and laboratory information. As diabetes mellitus was registered based on medical history or the use of anti-diabetic medication, the possibility of previously unrecognized or new-onset diabetes mellitus cannot be excluded and could lead to misclassification of diabetic status. The type of diabetes mellitus was not registered and we cannot provide information on diabetes mellitus control prior to ICU admission, because HbA1c was not routinely measured and diabetes mellitus care is usually provided by general practitioners. In addition, as plasma protein biomarkers were measured at intervals, relative rapid changes in biomarker distribution could not be analyzed.

## Conclusion

Neither diabetes mellitus nor preadmission insulin or metformin use are associated with altered disease severity, outcome or host response in patients with sepsis requiring intensive care.

## Abbreviations

APACHE-IV, Acute Physiology and Chronic health Evaluation IV; APS, Acute Physiology Score; aPTT, activated partial thromboplastin time; BH, Benjamini-Hochberg; BMI, body mass index; CCG, Cologne Center for Genomics; CRP, C-reactive protein; EDTA, ethylenediaminetetraacetic acid; HR, hazard ratio; ICU, intensive care unit; IL, interleukin; IPA, Ingenuity Pathway Analysis; IQR, interquartile ranges; MARS, Molecular Diagnosis and Risk Stratification of Sepsis; MMP-8, matrix metalloproteinase-8; PT, prothrombin time; RNA, ribonucleic acid; SD, standard deviation; sICAM-1, soluble intercellular adhesion molecule-1; SOFA, Sequential Organ Failure Assessment; TNF-α, tumor necrosis factor-α

## Appendix

### Total list of participating investigators in this multicenter study

Friso M. de Beer, M.D., Lieuwe D. J. Bos, M.D. Ph.D. (Department of Intensive Care, Academic Medical Center, University of Amsterdam), Marc M.J. Bonten M.D. PhD. (Department of Medical Microbiology, University Medical Center Utrecht), Olaf L. Cremer, M.D. PhD. (Department of Intensive Care Medicine, University Medical Center Utrecht), Jos F. Frencken, M.D. (Department of Intensive Care Medicine and Julius Center for Health Sciences and Primary Care, University Medical Center Utrecht), Gerie J. Glas, M.D., Roosmarijn T. M. van Hooijdonk, M.D. Ph.D. (Department of Intensive Care, Academic Medical Center, University of Amsterdam), Arie J. Hoogendijk PhD. (Center for Experimental and Molecular Medicine, Academic Medical Center, University of Amsterdam), Janneke Horn M.D. PhD. (Department of Intensive Care, Academic Medical Center, University of Amsterdam), Michaëla A.M. Huson, M.D. (Center for Experimental and Molecular Medicine, Academic Medical Center, University of Amsterdam), Peter M.C. Klein Klouwenberg M.D. PharmD. PhD., David S.Y. Ong, M.D. PharmD. (Department of Intensive Care Medicine, Department of Medical Microbiology and Julius Center for Health Sciences and Primary Care, University Medical Center Utrecht), Tom van der Poll M.D. PhD. (Center for Experimental and Molecular Medicine and Division of Infectious Diseases, Academic Medical Center, University of Amsterdam), Laura R. A. Schouten M.D. Marcus J. Schultz M.D. PhD., Marleen Straat (Department of Intensive Care, Academic Medical Center, University of Amsterdam), Brendon P. Scicluna PhD. Lonneke A van Vught, M.D. (Center for Experimental and Molecular Medicine, Academic Medical Center, University of Amsterdam), Esther Witteveen M.D. (Department of Intensive Care, Academic Medical Center, University of Amsterdam), Maryse A Wiewel M.D. (Center for Experimental and Molecular Medicine, Academic Medical Center, University of Amsterdam), Luuk Wieske, M.D. Ph.D. (Department of Intensive Care, Academic Medical Center, University of Amsterdam).

## Additional files

Additional file 1: Table S1. Plasma protein biomarkers at day 2 and 4 after intensive care unit admission of sepsis patients with or without diabetes metllitus. (DOC 57.5 kb) Additional file 2: Table S2. Baseline characteristics and outcome of sepsis patients with or without diabetes mellitus included in the whole genome-wide expression analysis. (DOC 82.5 kb) Additional file 3: Figure S1. Blood leukocyte genomic response in patients with diabetes mellitus and sepsis who were discordant for insulin or metformin treatment. Dot plots integrating log2-transformed fold changes in samples from (**A**) insulin-treated versus non-insulin-treated, and (**B**) metformin-treated versus non-metformin-treated patients with diabetes mellitus and sepsis. *Rho* Spearman’s correlation coefficient. (TIF 408 kb)

## References

[1] Angus DC, van der Poll T (2013). Severe sepsis and septic shock. N Engl J Med.

[2] Martin GS, Mannino DM, Eaton S, Moss M (2003). The epidemiology of sepsis in the United States from 1979 through 2000. N Engl J Med.

[3] Shah BR, Hux JE (2003). Quantifying the risk of infectious diseases for people with diabetes. Diabetes Care.

[4] Koh GC, Peacock SJ, van der Poll T, Wiersinga WJ (2012). The impact of diabetes on the pathogenesis of sepsis. Eur J Clin Microbiol Infect Dis.

[5] Lontchi-Yimagou E, Sobngwi E, Matsha TE, Kengne AP (2013). Diabetes mellitus and inflammation. Curr Diab Rep.

[6] Carr ME (2001). Diabetes mellitus: a hypercoagulable state. J Diabetes Complicat.

[7] Chang CW, Kok VC, Tseng TC, Horng JT, Liu CE (2012). Diabetic patients with severe sepsis admitted to intensive care unit do not fare worse than non-diabetic patients: a nationwide population-based cohort study. PLoS One.

[8] Schuetz P, Jones AE, Howell MD, Trzeciak S, Ngo L, Younger JG, Aird W, Shapiro NI (2011). Diabetes is not associated with increased mortality in emergency department patients with sepsis. Ann Emerg Med.

[9] Stegenga ME, Vincent JL, Vail GM, Xie J, Haney DJ, Williams MD, Bernard GR, van der Poll T (2010). Diabetes does not alter mortality or hemostatic and inflammatory responses in patients with severe sepsis. Crit Care Med.

[10] Vincent JL, Preiser JC, Sprung CL, Moreno R, Sakr Y (2010). Insulin-treated diabetes is not associated with increased mortality in critically ill patients. Crit Care.

[11] Schuetz P, Yano K, Sorasaki M, Ngo L, St Hilaire M, Lucas JM, Aird W, Shapiro NI (2011). Influence of diabetes on endothelial cell response during sepsis. Diabetologia.

[12] Dandona P, Aljada A, Mohanty P, Ghanim H, Hamouda W, Assian E, Ahmad S (2001). Insulin inhibits intranuclear nuclear factor kappaB and stimulates IkappaB in mononuclear cells in obese subjects: evidence for an anti-inflammatory effect?. J Clin Endocrinol Metab.

[13] Jeschke MG, Klein D, Bolder U, Einspanier R (2004). Insulin attenuates the systemic inflammatory response in endotoxemic rats. Endocrinology.

[14] Stegenga ME, van der Crabben SN, Blumer RM, Levi M, Meijers JC, Serlie MJ, Tanck MW, Sauerwein HP, van der Poll T (2008). Hyperglycemia enhances coagulation and reduces neutrophil degranulation, whereas hyperinsulinemia inhibits fibrinolysis during human endotoxemia. Blood.

[15] Stegenga ME, van der Crabben SN, Dessing MC, Pater JM, van den Pangaart PS, de Vos AF, Tanck MW, Roos D, Sauerwein HP, van der Poll T (2008). Effect of acute hyperglycaemia and/or hyperinsulinaemia on proinflammatory gene expression, cytokine production and neutrophil function in humans. Diabet Med.

[16] Isoda K, Young JL, Zirlik A, MacFarlane LA, Tsuboi N, Gerdes N, Schonbeck U, Libby P (2006). Metformin inhibits proinflammatory responses and nuclear factor-kappaB in human vascular wall cells. Arterioscler Thromb Vasc Biol.

[17] Kim J, Kwak HJ, Cha JY, Jeong YS, Rhee SD, Kim KR, Cheon HG (2014). Metformin suppresses lipopolysaccharide (LPS)-induced inflammatory response in murine macrophages via activating transcription factor-3 (ATF-3) induction. J Biol Chem.

[18] Vasamsetti SB, Karnewar S, Kanugula AK, Thatipalli AR, Kumar JM, Kotamraju S (2015). Metformin inhibits monocyte-to-macrophage differentiation via AMPK-mediated inhibition of STAT3 activation: potential role in atherosclerosis. Diabetes.

[19] Klein Klouwenberg PM, Ong DS, Bos LD, de Beer FM, van Hooijdonk RT, Huson MA, Straat M, van Vught LA, Wieske L, Horn J (2013). Interobserver agreement of Centers for Disease Control and Prevention criteria for classifying infections in critically ill patients. Crit Care Med.

[20] Scicluna BP, Klein Klouwenberg PM, van Vught LA, Wiewel MA, Ong DS, Zwinderman AH, Franitza M, Toliat MR, Nurnberg P, Hoogendijk AJ (2015). A molecular biomarker to diagnose community-acquired pneumonia on intensive care unit admission. Am J Respir Crit Care Med.

[21] van Vught LA, Klein Klouwenberg PM, Spitoni C, Scicluna BP, Wiewel MA, Horn J, Schultz MJ, Nurnberg P, Bonten MJ, Cremer OL, van der Poll T, Consortium M. Incidence, Risk Factors, and Attributable Mortality of Secondary Infections in the Intensive Care Unit After Admission for Sepsis. JAMA. 2016;315(14):1469–1479. 10.1001/jama.2016.2691 26975785

[22] Levy MM, Fink MP, Marshall JC, Abraham E, Angus D, Cook D, Cohen J, Opal SM, Vincent JL, Ramsay G (2003). 2001 SCCM/ESICM/ACCP/ATS/SIS International Sepsis Definitions Conference. Crit Care Med.

[23] Charlson ME, Pompei P, Ales KL, MacKenzie CR (1987). A new method of classifying prognostic comorbidity in longitudinal studies: development and validation. J Chronic Dis.

[24] Zimmerman JE, Kramer AA, McNair DS, Malila FM (2006). Acute Physiology and Chronic Health Evaluation (APACHE) IV: hospital mortality assessment for today’s critically ill patients. Crit Care Med.

[25] Vincent JL, Moreno R, Takala J, Willatts S, De Mendonca A, Bruining H, Reinhart CK, Suter PM, Thijs LG (1996). The SOFA (Sepsis-related Organ Failure Assessment) score to describe organ dysfunction/failure. On behalf of the Working Group on Sepsis-Related Problems of the European Society of Intensive Care Medicine. Intensive Care Med.

[26] Kaukonen KM, Bailey M, Suzuki S, Pilcher D, Bellomo R (2014). Mortality related to severe sepsis and septic shock among critically ill patients in Australia and New Zealand, 2000-2012. JAMA.

[27] Bellomo R, Ronco C, Kellum JA, Mehta RL, Palevsky P, Acute Dialysis Quality Initiative (2004). Acute renal failure - definition, outcome measures, animal models, fluid therapy and information technology needs: the Second International Consensus Conference of the Acute Dialysis Quality Initiative (ADQI) Group. Crit Care.

[28] Force ADT, Ranieri VM, Rubenfeld GD, Thompson BT, Ferguson ND, Caldwell E, Fan E, Camporota L, Slutsky AS (2012). Acute respiratory distress syndrome: the Berlin Definition. JAMA.

[29] Bourgon R, Gentleman R, Huber W (2010). Independent filtering increases detection power for high-throughput experiments. Proc Natl Acad Sci USA.

[30] Johnson WE, Li C, Rabinovic A (2007). Adjusting batch effects in microarray expression data using empirical Bayes methods. Biostatistics.

[31] Smyth GK. Limma: linear models for microarray data. Bioinformatics and Computational Biology Solutions using R; Springer. 2005. p. 397–420.

[32] R Core Team. R: A language and environment for statistical computing. R Foundation for Statistical Computing; 2013. Available from: http://www.r-project.org/

[33] Kornum JB, Thomsen RW, Riis A, Lervang HH, Schonheyder HC, Sorensen HT (2007). Type 2 diabetes and pneumonia outcomes: a population-based cohort study. Diabetes Care.

[34] Thomsen RW, Hundborg HH, Lervang HH, Johnsen SP, Schonheyder HC, Sorensen HT (2005). Diabetes mellitus as a risk and prognostic factor for community-acquired bacteremia due to enterobacteria: a 10-year, population-based study among adults. Clin Infect Dis.

[35] Graham BB, Keniston A, Gajic O, Trillo Alvarez CA, Medvedev S, Douglas IS (2010). Diabetes mellitus does not adversely affect outcomes from a critical illness. Crit Care Med.

[36] Yende S, van der Poll T, Lee M, Huang DT, Newman AB, Kong L, Kellum JA, Harris TB, Bauer D, Satterfield S (2010). The influence of pre-existing diabetes mellitus on the host immune response and outcome of pneumonia: analysis of two multicentre cohort studies. Thorax.

[37] Opal SM, van der Poll T (2015). Endothelial barrier dysfunction in septic shock. J Intern Med.

[38] Bertoluci MC, Ce GV, da Silva AM, Wainstein MV, Boff W, Punales M (2015). Endothelial dysfunction as a predictor of cardiovascular disease in type 1 diabetes. World J Diabetes.

[39] Morigi M, Angioletti S, Imberti B, Donadelli R, Micheletti G, Figliuzzi M, Remuzzi A, Zoja C, Remuzzi G (1998). Leukocyte-endothelial interaction is augmented by high glucose concentrations and hyperglycemia in a NF-kB-dependent fashion. J Clin Invest.

[40] Duncan AI, Koch CG, Xu M, Manlapaz M, Batdorf B, Pitas G, Starr N (2007). Recent metformin ingestion does not increase in-hospital morbidity or mortality after cardiac surgery. Anesth Analg.

[41] Christiansen C, Johansen M, Christensen S, O’Brien JM, Tonnesen E, Sorensen H (2013). Preadmission metformin use and mortality among intensive care patients with diabetes: a cohort study. Crit Care.

