# Supplementary material for: Association of diabetes and diabetes treatment with the host response in critically ill sepsis patients
Source: Crit Care. 2016 Aug 6;20:252. doi: 10.1186/s13054-016-1429-8 (PMC4975896; doi:10.1186/s13054-016-1429-8)
Supplement: Additional file 1: Table S1. — Plasma protein biomarkers at day 2 and 4 after intensive care unit admission of sepsis patients with or without diabetes metllitus. (DOC 57.5 kb) [file 13054_2016_1429_MOESM1_ESM.doc]

**Supplemental file**

**Association of diabetes and diabetes treatment with the host response in critically ill sepsis patients**

Lonneke A. van Vught, M.D.^1,2^, Brendon P. Scicluna, PhD^1,2^, Arie J. Hoogendijk, PhD^1,2^, Maryse A. Wiewel, M.D.^1,2^, Peter M.C. Klein Klouwenberg, M.D., PharmD. PhD.^3,4,5^, Olaf L. Cremer, M.D., PhD^3^, Janneke Horn, M.D., PhD^6^, Peter Nürnberg^7,8,9^, Marc M.J. Bonten, M.D., PhD^4^, Marcus J. Schultz, M.D., PhD^6,10^ and Tom van der Poll, M.D., PhD^1,2,11^

^1^Center for Experimental and Molecular Medicine, Academic Medical Center, University of Amsterdam, Amsterdam, the Netherlands; ^2^the Center for Infection and Immunity, Academic Medical Center, University of Amsterdam, Amsterdam, the Netherlands; ^3^Department of Intensive Care Medicine, University Medical Center Utrecht, Utrecht, the Netherlands; ^4^Department of Medical Microbiology, University Medical Center Utrecht, Utrecht, the Netherlands; ^5^Julius Center for Health Sciences and Primary Care, University Medical Center Utrecht, Utrecht, the Netherlands; ^6^Department of Intensive Care, Academic Medical Center, University of Amsterdam, Amsterdam, the Netherlands. ^7^Cologne Center for Genomics (CCG), University of Cologne, Cologne, Germany; ^8^Cologne Excellence Cluster on Cellular Stress Responses in Aging-Associated Diseases (CECAD), University of Cologne, Cologne, Germany; ^9^Center for Molecular Medicine Cologne (CMMC), University of Cologne, Cologne, Germany; ^10^ Laboratory of Experimental Intensive Care and Anesthesiology, Academic Medical Center, University of Amsterdam, Amsterdam, the Netherlands; ^11^Division of Infectious Diseases, Academic Medical Center, University of Amsterdam, Amsterdam, the Netherlands.

**Table S1. Plasma biomarkers at day 2 and 4 after intensive care unit admission of sepsis patients with or without diabetes**

|  | **Day 2**^†^ | | **Day 4**^†^ | |
| --- | --- | --- | --- | --- |
|  | **Diabetes** | **No Diabetes** | **Diabetes** | **No Diabetes** |
| **Inflammatory and cytokine responses** | N = 162 | N = 596 | N = 112 | N = 416 |
| CRP (mg/ml) | 217 [131-339] | 218 [130-324] | 99 [67-178] | 128 [66-223] |
| IL-6 (pg/ml) | 58.73 [21.51-150.16] | 71.79 [25.55-276.5] | 40.26 [13.5-98.8] | 38.48 [14.69-118.17] |
| IL-8 (pg/ml) | 67.78 [30.39-144.82] | 77.47 [28.09-222.48] | 55.25 [25.57] | 62.43 [21.53-160.71] |
| IL-10 (pg/ml) | 6.99 [3.19-13.91] | 6.87 [2.86-21.56] | 4.45 [2.29-15.48] | 5.06 [2.32-12.64] |
| MMP-8 (ng/mL) | 2.36 [0.67-6.61] | 2.37 [0.86-8.12] | 1.23 [0.47-3.35] | 1.55 [0.58-5.03] |
| **Procoagulang response** |  |  |  |  |
| D-dimer (µg/ml) | 8.26 [3.15-16.95] | 9.74 [4.61-17.96] | 9.30 [5.68-16.25] | 10.62 [5.26-19.22] |
| Prothrombin time | 15.2 [12.8-17.9] | 14.7 [12.5-17.2] | 14.6 [12.6-16.3] | 13.9 [11.9-15.8] |
| aPTT | 40.5 [31-57.8] | 39.0 [32-50] | 37 [30-50.8] | 37.0 [28-48] |
| Protein C (ng/ml) | 118.48 [88.39-158.8] | 122.04 [89.61-160.87] | 133.71 [99.42-167.59] | 130.69 [91.27-186.12] |
| Antithrombin (ng/ml) | 731.15 [448.65-1159.25] | 694.86 [438.27-1033.33] | 825.12 [617.24-1284.92] | 853.37 [529.5-1361.28] |
| **Endothelial cell activation** |  |  |  |  |
| sE-Selectin (ng/ml) | 9.18 [4.31-18.19] | 9.25 [4.23-19.54] | 8.12 [3.73-12.97] | 7.72 [3.98-14.68] |
| sICAM-1 (ng/ml) | 208.76 [113.4-331.8] | 208.03 [119.85-344.23] | 232.86 [138.45-356.67] | 211.16 [127.93-343.73] |
| Fractalkine | 27.87 [17.75-48.09] | 24.07 [17.54-56.22] | 29.82 [18.22-63.84] | 27.08 [17.79-53.53] |
| Ang-1 (ng/ml) | 1.71 [0.76-4.26] | 1.50 [0.74-3.5] | 1.71 [0.65-3.82] | 1.43 [0.62-3.57] |
| Ang-2 (ng/ml) | 7.78 [2.52-18.54] | 8.33 [3.58-19.47] | 6.60 [2.6-11.7] | 5.82 [2.72-14.5] |

Plasma levels on day 2 and 4 after intensive care unit admission. Results are presented as medians and interquartile ranges.

Abbreviations: CRP: C-reactive protein , IL: interleukin, MMP: matrix metalloproteinase, aPPT: activated partial thromboplastin time, sICAM: soluble intercellular adhesion molecule, ANG: angiopoietin.

^†^ Bonferoni corrected p-values were not different between diabetes and non-diabetes patients on day 2 or day 4 using a Mann-Whitney *U* test for non-parametric data
