# Supplementary material for: Association of diabetes and diabetes treatment with the host response in critically ill sepsis patients
Source: Crit Care. 2016 Aug 6;20:252. doi: 10.1186/s13054-016-1429-8 (PMC4975896; doi:10.1186/s13054-016-1429-8)
Supplement: Additional file 2: Table S2. — Baseline characteristics and outcome of sepsis patients with or without diabetes mellitus included in the whole genome-wide expression analysis. (DOC 82.5 kb) [file 13054_2016_1429_MOESM2_ESM.doc]

**Supplemental file**

**Association of diabetes and diabetes treatment with the host response in critically ill sepsis patients**

Lonneke A. van Vught, M.D.^1,2^, Brendon P. Scicluna, PhD^1,2^, Arie J. Hoogendijk, PhD^1,2^, Maryse A. Wiewel, M.D.^1,2^, Peter M.C. Klein Klouwenberg, M.D., PharmD. PhD.^3,4,5^, Olaf L. Cremer, M.D., PhD^3^, Janneke Horn, M.D., PhD^6^, Peter Nürnberg^7,8,9^, Marc M.J. Bonten, M.D., PhD^4^, Marcus J. Schultz, M.D., PhD^6,10^ and Tom van der Poll, M.D., PhD^1,2,11^

^1^Center for Experimental and Molecular Medicine, Academic Medical Center, University of Amsterdam, Amsterdam, the Netherlands; ^2^the Center for Infection and Immunity, Academic Medical Center, University of Amsterdam, Amsterdam, the Netherlands; ^3^Department of Intensive Care Medicine, University Medical Center Utrecht, Utrecht, the Netherlands; ^4^Department of Medical Microbiology, University Medical Center Utrecht, Utrecht, the Netherlands; ^5^Julius Center for Health Sciences and Primary Care, University Medical Center Utrecht, Utrecht, the Netherlands; ^6^Department of Intensive Care, Academic Medical Center, University of Amsterdam, Amsterdam, the Netherlands. ^7^Cologne Center for Genomics (CCG), University of Cologne, Cologne, Germany; ^8^Cologne Excellence Cluster on Cellular Stress Responses in Aging-Associated Diseases (CECAD), University of Cologne, Cologne, Germany; ^9^Center for Molecular Medicine Cologne (CMMC), University of Cologne, Cologne, Germany; ^10^ Laboratory of Experimental Intensive Care and Anesthesiology, Academic Medical Center, University of Amsterdam, Amsterdam, the Netherlands; ^11^Division of Infectious Diseases, Academic Medical Center, University of Amsterdam, Amsterdam, the Netherlands.

**Table S2. Baseline characteristics and outcome** of sepsis patients with or without diabetes included in the whole genome wide expression analysis

|  | | | **Diabetes** | **No diabetes** | **P value** |
| --- | --- | --- | --- | --- | --- |
| **Patients** | | | 108 | 382 |  |
| **Demographics** | | |  |  |  |
| Age. mean (SD) | | | 67.0 (11.6) | 60.5 (15.3) | <.0001 |
| Gender male, n (%) | | | 61 (56.5%) | 223 (58.4%) | 0.73 |
| BMI mean (SD) | | | 28.7 (8.1) | 25.5 (5.8) | <.001 |
| Race, white, n (%) | | | 93 (86.1%) | 329 (86.1%) | 0.76 |
| Medical admission, n (%) | | | 85 (78.7%) | 270 (70.7%) | 0.12 |
| **Chronic comorbidity, n (%)** | | |  |  |  |
|  | Cardiovascular compromise | | 44 (40.7%) | 55 (14.4%) | <.001 |
|  | COPD | | 17 (15.7%) | 51 (13.4%) | 0.55 |
|  | Hypertension | | 54 (50.0%) | 89 (23.3%) | <.001 |
|  | Malignancy | | 25 (23.1%) | 90 (23.6%) | >.99 |
|  | Renal insufficiency | | 24 (22.2%) | 49 (12.8%) | 0.02 |
|  | Modified Charlson comorbidity index* | | 5 [3-6] | 4 [2-5] | 0.0001 |
| **Diabetic medication, n (%)** | | |  |  |  |
|  | Insulin | | 57 (52.8%) | - |  |
|  | Metformin | | 55 (50.9%) | - |  |
|  | Glimepiride | | 10 (9.3%) | - |  |
|  | Glibenclamide | | 10 (9.3%) | - |  |
|  | Gliclazide | | 3 (2.8%) | - |  |
|  | Tolbutamide | | 8 (7.4%) | - |  |
| **Severity of disease on ICU admission** | | |  |  |  |
|  | APACHE IV Score, median [IQR] | | 82 [65-101] | 81 [65-100] | 0.63 |
|  | APACHE APS, median [IQR] | | 66 [50-84] | 67 [53-85] | 0.59 |
|  | SOFA score, median [IQR] | | 7 [5-9] | 7 [5-10] | 0.97 |
|  | Mechanical ventilation, n (%) | | 71 (65.7%) | 278 (72.8%) | 0.18 |
|  | Organ failure, n (%) | | 91 (84.3%) | 322 (84.3%) | 0.82 |
|  | Shock, n (%) | | 39 (36.1%) | 140 (36.6%) | >.99 |
|  | Acute kidney injury, n (%) | | 44 (40.7%) | 135 (35.3%) | 0.29 |
|  | Acute lung injury, n (%) | | 34 (31.5%) | 111 (29.1%) | 0.66 |
|  | Acute myocardial infarction, n (%) | | 2 (1.9%) | 5 (1.3%) | >.99 |
| **Outcome** | | |  |  |  |
|  | ICU Length of stay (days), median [IQR] | | 4 [2-10] | 5 [2-10] | 0.58 |
|  | Hospital length of stay (days), median [IQR] | | 21 [11-44] | 23 [13-47] | 0.23 |
|  | Complications, n (%) | |  |  |  |
|  |  | None | 88 (81.5%) | 304 (79.6%) | 0.70 |
|  |  | Acute kidney injury | 13 (12.0%) | 31 (8.1%) | 0.25 |
|  |  | Acute lung injury | 3 (2.8%) | 19 (5.0%) | 0.43 |
|  |  | ICU-acquired weakness | 8 (7.4%) | 35 (9.2%) | 0.69 |
|  |  | Acute myocardial infarction | - | 3 (0.8%) | 0.60 |
|  |  | ICU-acquired infection | 9 (8.3%) | 38 (9.9%) | 0.73 |
| **Mortality, n (%)** | | |  |  |  |
|  | ICU | | 20 (18.5%) | 76 (19.9%) | 0.79 |
|  | Hospital | | 35 (32.4%) | 117 (30.6%) | 0.80 |
|  | Day 30 | | 30 (27.8%) | 103 (27.0%) | 0.90 |
|  | Day 60 | | 42 (38.9%) | 118 (30.9%) | 0.13 |
|  | Day 90 | | 44 (40.7%) | 133 (34.8%) | 0.25 |

***** Modified Charlson is calculated without the contribution of diabetes.

Abbreviations: APACHE: Acute Physiology and Chronic Health Evaluation; APS: Acute Physiology Score; BMI: Body Mass Index; COPD: Chronic Obstructive Pulmonary Disease; ICU: Intensive Care Unit.
