# Supplementary figures and images for: Association of diabetes and diabetes treatment with the host response in critically ill sepsis patients
Source: Crit Care. 2016 Aug 6;20:252. doi: 10.1186/s13054-016-1429-8 (PMC4975896; doi:10.1186/s13054-016-1429-8)

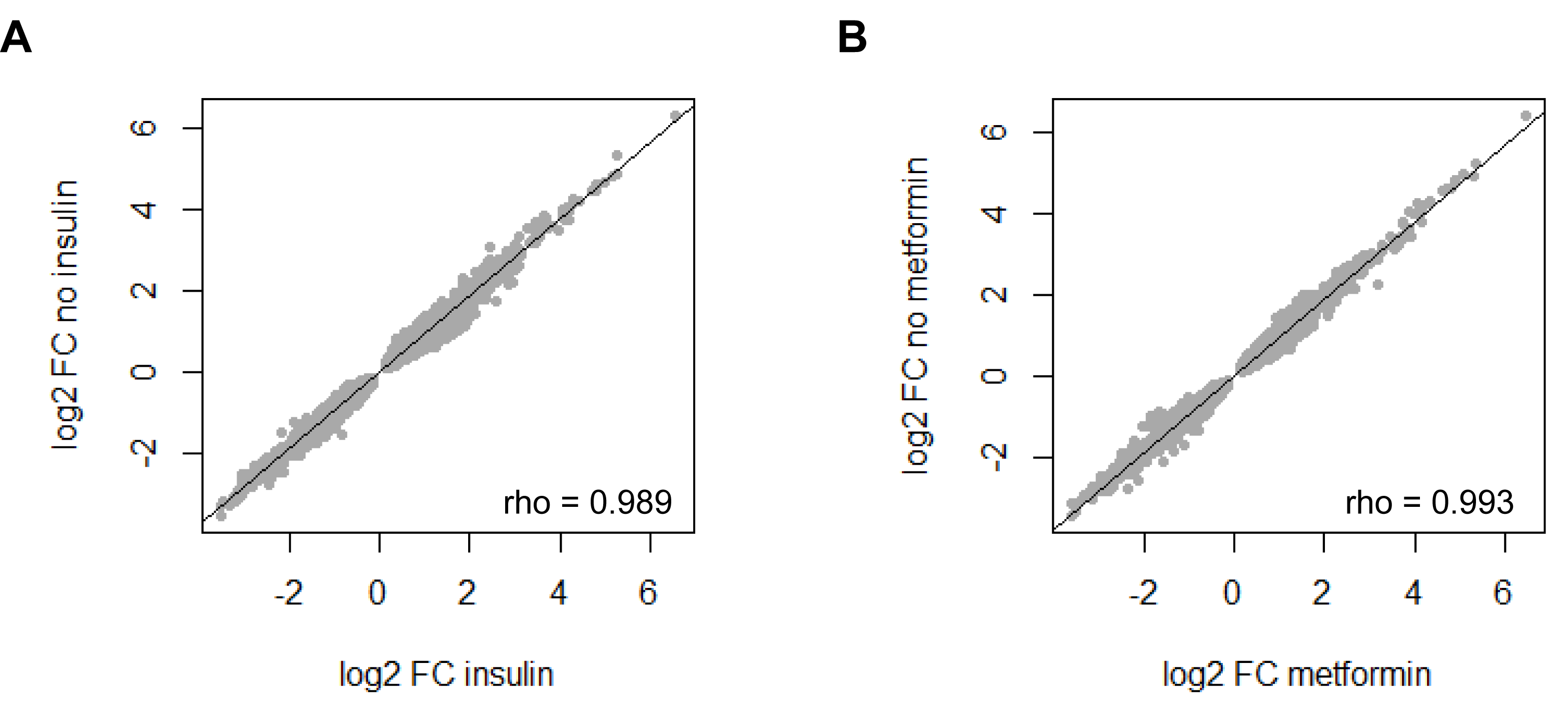

Supplement: Additional file 3: Figure S1. — Blood leukocyte genomic response in patients with diabetes mellitus and sepsis who were discordant for insulin or metformin treatment. Dot plots integrating log2-transformed fold changes in samples from (A) insulin-treated versus non-insulin-treated, and (B) metformin-treated versus non-metformin-treated patients with diabetes mellitus and sepsis. Rho Spearman’s correlation coefficient. (TIF 408 kb) [file 13054_2016_1429_MOESM3_ESM.tif]
